# Pdx1 Is Post-Translationally Modified *In vivo* and Serine 61 Is the Principal Site of Phosphorylation

**DOI:** 10.1371/journal.pone.0035233

**Published:** 2012-04-11

**Authors:** Thomas Frogne, Kathrine Beck Sylvestersen, Stefan Kubicek, Michael Lund Nielsen, Jacob Hecksher-Sørensen

**Affiliations:** 1 Department of Beta-cell Regeneration, Hagedorn Research Institute, Gentofte, Denmark; 2 Center for Protein Research, University of Copenhagen, Copenhagen, Denmark; 3 Research Center for Molecular Medicine of the Austrian Academy of Sciences, Vienna, Austria; University of Tor Vergata, Italy

## Abstract

Maintaining sufficient levels of Pdx1 activity is a prerequisite for proper regulation of blood glucose homeostasis and beta cell function. Mice that are haploinsufficient for Pdx1 display impaired glucose tolerance and lack the ability to increase beta cell mass in response to decreased insulin signaling. Several studies have shown that post-translational modifications are regulating Pdx1 activity through intracellular localization and binding to co-factors. Understanding the signaling cues converging on Pdx1 and modulating its activity is therefore an attractive approach in diabetes treatment. We employed a novel technique called Nanofluidic Proteomic Immunoassay to characterize the post-translational profile of Pdx1. Following isoelectric focusing in nano-capillaries, this technology relies on a pan specific antibody for detection and it therefore allows the relative abundance of differently charged protein species to be examined simultaneously. In all eukaryotic cells tested we find that the Pdx1 protein separates into four distinct peaks whereas Pdx1 protein from bacteria only produces one peak. Of the four peaks in eukaryotic cells we correlate one of them to a phosphorylation Using alanine scanning and mass spectrometry we map this phosphorylation to serine 61 in both Min6 cells and in exogenous Pdx1 over-expressed in HEK293 cells. A single phosphorylation is also present in cultured islets but it remains unaffected by changes in glucose levels. It is present during embryogenesis but is not required for pancreas development.

## Introduction

*Pancreatic and duodenal homeobox 1* (*Pdx1*) also known as *Ipf1* and *Stf1* is a master regulator of pancreas development [1], [2], [3]. *Pdx1* was first cloned and described in *Xenopus*, as *Xlhbox8*, where it was shown to be expressed in the developing endoderm and pancreas [4]. In mouse embryos *Pdx1* is expressed in the endoderm from e8.5 where it defines the regions that will form the dorsal and ventral pancreas [1], [2], [5]. The evidence that *Pdx1* is instrumental for pancreas development comes from both mouse and human where depletion of a functional Pdx1 protein results in pancreas agenesis [1], [2], [6]. Conversely, over expression of Pdx1 in endodermal cells outside the presumptive pancreas can activate events reminiscent of pancreas development. In chicken embryos forced expression of Pdx1 in the developing endoderm partially induces pancreas development. Thus, ectopic Pdx1 quenches the expression of non-pancreatic genes such as *Sox2* and *Cdx* in regions outside the presumptive pancreas [7] while it induces pancreatic markers like *Nkx6*.*1* [8]. In *Xenopus*, over expression of a modified *Pdx1*, carrying the VP16 transcriptional activation domain can cause conversion of liver to pancreas [9].

Postnatally *Pdx1* is expressed in the mature β-cell where it serves as an important regulator of glucose homeostasis [10], [11]. In humans, mutations in the *Pdx1* gene have been associated with type 2 diabetes and maturity onset diabetes of the young 4 (MODY4) [12], [13]. This role is conserved in evolution and impaired glucose tolerance has been observed in several animal models where Pdx1 protein levels have been depleted or reduced [10], [14], [15], [16], [17], [18]. Furthermore, the diabetic phenotype observed following Pdx1 inactivation is reversible and blood glucose levels can be normalized if *Pdx1* expression is reactivated [19]. In the sand rat (*Psammonys obesus*) which normally lacks Pdx1 in the β-cells, reintroduction of the protein greatly improves glucose stimulated insulin transcription [15]. Several studies have shown that Pdx1 directly influences glucose homeostasis by binding and regulating the promoters of genes such as *insulin* (*ins*), *glut2* (*Slc2a2*) and *glucokinase* (*Gck*) [3], [20], [21], [22]. However, in addition to the immediate effects of Pdx1 on glucose homeostasis studies in mice haploinsufficient for *Pdx1* have revealed a long term requirement for correct Pdx1 dosage. In the mature β-cell the loss of one *Pdx1* allele affects both glucose stimulated insulin release and β-cell survival [11]. Furthermore, the compensatory increase in β-cell mass associated with impaired insulin signaling relies on Pdx1 dosage. Mice that are double heterozygous for mutations in the *insulin receptor* (*Ir*) and the *insulin receptor substrate* (*Irs*) display impaired insulin signaling and respond by increasing their the β-cell mass. This expansion is abolished in mice that are haploinsufficient for Pdx1 [23]. It is unclear why reductions in Pdx1 protein levels has such deteriorating effects on the β-cell, however, a recent study suggests that it may be linked to mitochondrial dysfunction. Thus preventing opening of the mitochondrial permeability transition pore through deletion of the *Peptidyl-prolyl cis-trans isomerase* (*Ppif*) gene restores many of the defects caused by Pdx1 insufficiency [24].

Since even subtle changes in Pdx1 levels dramatically impacts β-cell performance it is generally acknowledged that the biological activity of Pdx1 tightly regulated at the post-translational level. Fitting this hypothesis at least three different post-translational modifications have been reported on Pdx1 including phosphorylation [25], [26], [27], [28], [29], [30], [31], [32], [33], [34], [35], glycosylation [36] and sumoylation [37]. Phosphorylation, in particular, has been shown to regulate several important aspects of Pdx1 activity in response to elevated glucose levels, such as nuclear-cytoplasmic shuttling [26], [27], DNA binding [27], [28], [38] and interactions with transcriptional co-factors [25], [39]. Several different kinases have been shown to phosphorylate Pdx1 and to date five different residues have been suggested to be Pdx1 kinase substrates. Amino acids S61 or S66 are phosphorylated by Glycogen synthase kinase 3 (Gsk3) and ERK1/2 [29], [31] and this modification targets Pdx1 for proteasomal degradation in response to oxidative stress [29]. Amino acids S268 and S272 (S269 and S273 in mouse) are phosphorylated by Hipk2 [40] or in response to low glucose [30] and T11 is phosphorylated by DNA-dependent kinase in response to DNA damage [33]. Yet, despite the attention several important questions remain unanswered. For example it is unclear to what extent Pdx1 is modified under normal conditions? How many PTMs are present at a given time? How do different PTMs influence each other? How is the endogenous Pdx1 modified in respond to various treatments?

Here we have employed a novel technique called nanofluidic proteomic immunoassay (NIA) [41], [42] to study post-translational modifications of Pdx1 in various cell types. NIA employs isoelectric focusing to separate differently charged protein species of the same protein which is then detected using a pan-specific antibody. A particular advantage of this technology is that experiments can be carried out from scarce samples [42]. We have therefore been able to identify and study different protein species of endogenous Pdx1 in cell lines, during development and in mature β-cells. In eukaryotic cells we observe that the Pdx1 protein separates into four peaks, while Pdx1 purified from bacteria only produces a single peak. There is no apparent difference in SDS-PAGE mobility between mammalian and bacterial Pdx1 proteins and we therefore conclude that the mammalian profile is the result of post-translational modifications. Treating the Pdx1 protein with phosphatase revealed the presence of one primary phosphorylation which was mapped to serine 61 using both alanine scanning and mass spectrometry. Remarkably, Pdx1 protein obtained from, embryonic tissue, adult pancreas, isolated islets, βTC cells, Min6 cells and from Pdx1 over-expressed in αTC, L and HEK293 cells produces almost identical profiles. Since the single phosphorylation is present in all cells and tissues tested we speculate that it is mediated by a ubiquitously expressed kinase rather than a specific signaling pathway. In line with this we were unable to demonstrate any alterations in the Pdx1 profile following high or low glucose treatment of mouse islets in culture. And although we confirm that Pdx1 is phosphorylated during embryonic pancreas development, we find that Pdx1^S61A^ is fully capable of inducing ectopic Nkx6.1 expression and budding of pancreatic like structures.

## Results

### Over Expressed Pdx1 Appear as Multiple Protein Species

**Figure 1:**
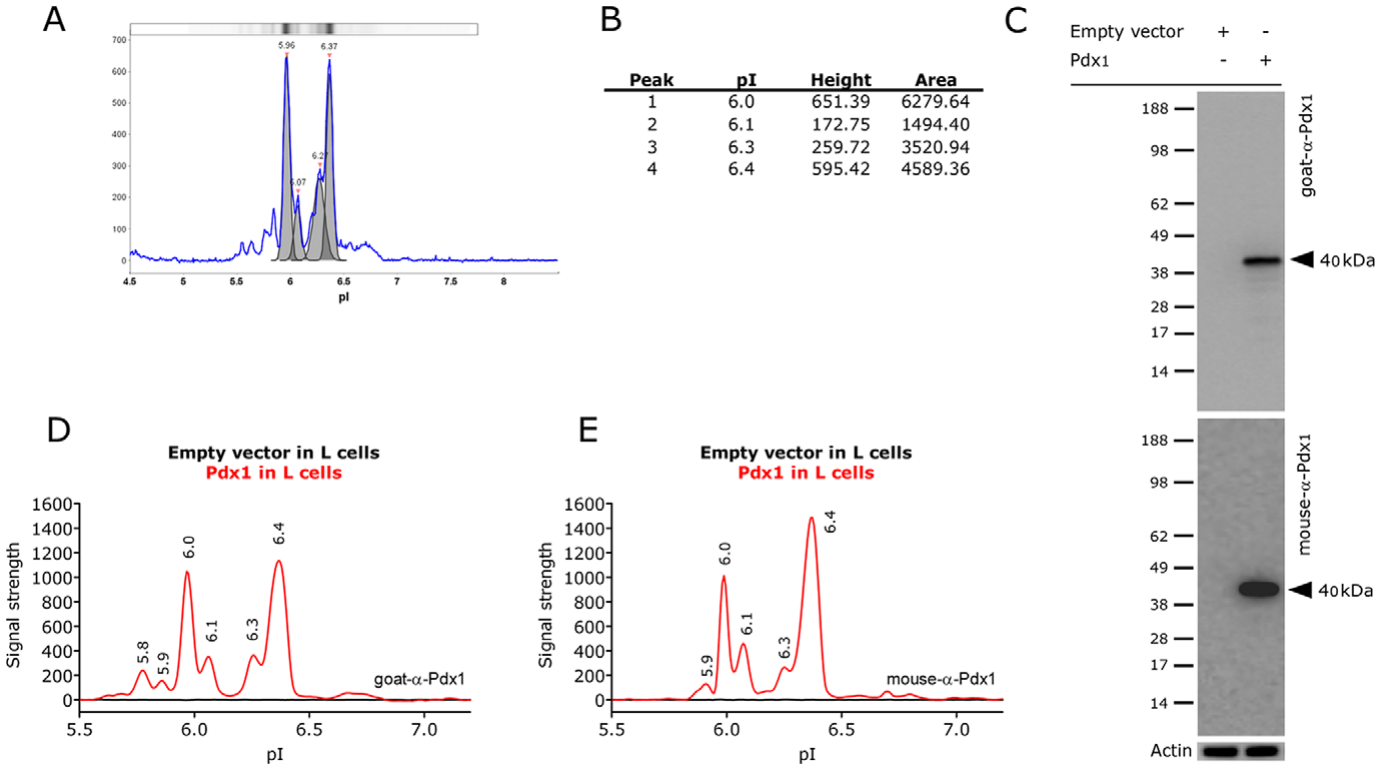
Over expression of Pdx1 in L and HEK293 cells. A) Raw data from the NIA analysis can be visualized as gel-bands or as a graph. B) The intensity of the signal can be estimated based on the height of the peak or the area under the curve. C-E) Over expression of empty vector or pdx1^WT^ in L cells followed by western blotting using a goat-α-Pdx1 (C, top) or a mouse-α-Pdx1 antibody (C, bottom) and NIA analysis of the same lysates using the goat-α-Pdx1 (D) or mouse-α-Pdx1 antibody (E).

To investigate the extent of post-translational modifications on the endogenous Pdx1 protein we applied a novel technology called NIA [41], [42]. Isoelectric focusing is used to separate differently charged species of a protein which subsequently can be detected using a pan-specific antibody that recognizes these species with similar affinity (assuming equal antibody binding to the different protein species). The raw data can be examined as bands on a gel or as peaks (Fig. 1A) where the relative abundance of the different protein species can be estimated from the height of the peaks or as the area under the curves (Fig. 1B). To precisely determine which peaks can be assigned to Pdx1 we performed western blot and NIA analysis on lysates from cells over-expressing wild type mouse Pdx1 (Pdx1^WT^), using two different antibodies against Pdx1. A polyclonal goat-α-Pdx1 antibody and a monoclonal mouse-α-Pdx1 antibody [43]. When used for western we found that both antibodies specifically detected a 40 kDa band in cells transfected with Pdx1^WT^ (Fig. 1C). When the same lysates was used for NIA the empty vector control displayed no peaks whereas both antibodies produced peaks at pI 5.9, 6.0, 6.1, 6.3 and 6.4 in Pdx1 transfected cells (Fig. 1D and E). The only difference was the 5.8 peak which is absent when using the mouse antibody.

### Pdx1 is Subject to Post-translational Modification in Mammalian Cells

**Figure 2:**
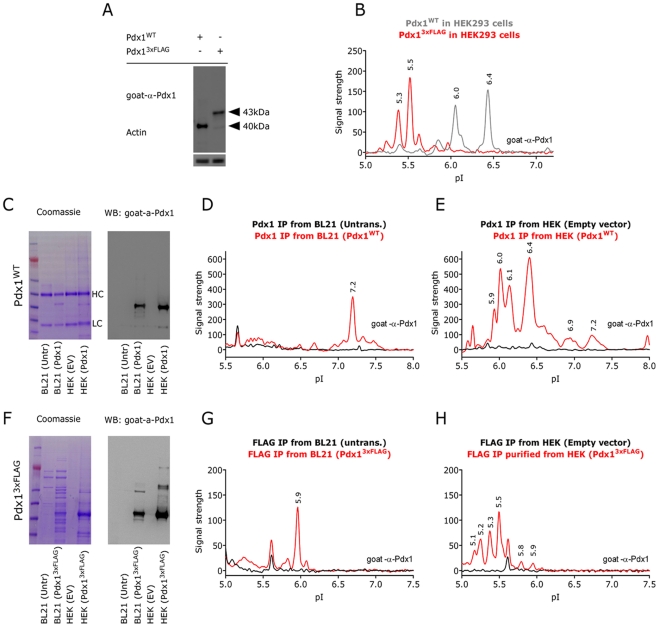
The Pdx1 NIA profile differs between bacterial and mammalian cells. First, a comparison of the basal NIA profile of pdx1^WT^ and pdx1^3xFLAG^ protein from transfected HEK293 cells by western blotting (A) and NIA (B), employing the goat-α-Pdx1 antibody. The amino acids in the 3xFLAG tag are negatively charged and in agreement a left shift of the NIA profile is observed. Results are representative of at least three independent experiments. Then a comparison of purified Pdx1^WT^ and Pdx1^3xFLAG^ protein from E.Coli BL21 cells versus purified protein from HEK293 cells. Plasmids coding for the indicated proteins were expressed in BL21or HEK293 cells and purified by immunoprecipitation using a monoclonal mouse-α-Pdx1 antibody coupled to protein G beads (Pdx1^WT^) or with anti-FLAG beads (Pdx1^3xFLAG^). As negative control cell lysate from non transformed bacteria or empty vector (EV) transfected HEK293 cells were ran in parallel. Purified protein from Pdx1^WT^ expressing and non expressing BL21 and HEK293 cells were analyzed by coomassie staining and western blot (C) and by NIA, (D) BL21, (E) HEK293. F-H) Similar analysis for Pdx1^3xFLAG^. These experiments were performed at least twice obtaining similar results.

To test if the antibodies specifically recognize various protein species of Pdx1 the entire profile should be shifted if the overall pI is changed. We therefore added a negatively charged 3xFLAG epitope tag to the C-terminus of wild type Pdx1 (Pdx1^3xFLAG^). This changes the predicted pI of Pdx1 from 6.4 for the wild type to 5.5 for the 3xFLAG tagged Pdx1. The increase in size was confirmed on a western blot (Fig. 2A) and as predicted the NIA profile of FLAG tagged Pdx1 was shifted to the left (Fig. 2B; red line). Importantly, all peaks are shifted to the left indicating that the added FLAG tag affects all Pdx1 protein species. It should be noted that the leftward shift of the profile is associated with a compression of the NIA profile, leading to the 6.0 and 6.1 as well as the 6.3 and 6.4 peaks becoming indistinguishable. However, to confirm that Pdx1 is actually modified we went on to purify Pdx1 from either bacteria or mammalian cells and analyzed the purified proteins using SDS-PAGE and NIA. The cDNA encoding wild type Pdx1 was cloned into two vectors which allowed expression in either bacteria or mammalian cells, respectively. Pdx1 protein was subsequently purified using the mouse-α-Pdx1 antibody for immunoprecipitation (IP) and then the goat-α-Pdx1 antibody for western and NIA. On SDS-PAGE the purified protein migrated at approximately 40 kDa and could be detected by western from both bacteria and HEK293 cells while control samples where negative (Fig. 2C). When the same samples were analyzed on NIA bacterial Pdx1 gave a single peak at pI 7.2 (Fig. 2D) while the profile obtained from mammalian Pdx1 was similar to that obtained without IP and the most prominent peaks were detected at pI 5.9, 6.0, 6.1, 6.4 but we also observed lower intensity peaks at pI 5.6, 6.5, 6.9 and 7.2 (Fig. 2E). To further validate these results we repeated the experiment using the Pdx1^3xFLAG^ construct. When this approach was used to purify protein from bacteria we detected some unspecific bands both using coomassie and western blotting (Fig. 2F), importantly, the Pdx1^3xFLAG^ band was the strongest signal in the lane and the controls were blank. NIA analysis of Pdx1^3xFLAG^ obtained from bacteria revealed one Pdx1 specific peak at pI 5.9, while a smaller 5.6 peak was present in both Pdx1 and control lysates (Fig. 2G). We therefore believe that the 5.9 peak is the Pdx1^3xFLAG^ protein. NIA analysis of Pdx1^3xFLAG^ purified from HEK cells resulted in four specific peaks at 5.1, 5.2, 5.3 and 5.5 which are similar to those observed when analyzing the lysates without the IP step (Fig. 2H). As for IP of Pdx1^WT^, lower intensity bands is also evident for Pdx1^3xFLAG^ and we believe that the peaks at pI 5.8 and 5.9 corresponds to the 6.9 and 7.2 peaks found for Pdx1^WT^. In contrast the 5.6 peak is found in both control and Pdx1 expressing cells and this peak does not move in the response to the 3xFLAG tag, indicating that it is unspecific binding of the goat-α-Pdx1 antibody.

Lastly, to rule out that differences in buffer composition following the IP could influence the migration of Pdx1 in the nano capillaries we carried out a series of experiments where the purified Pdx1 from bacteria or mammalian cells where spiked directly into lysates from HEK cells transfected with EGFP or Pdx1^3xFLAG^ (Fig. S1). These results strongly indicate that the bacterial Pdx1 generates one single peak with a pI that is different from the Pdx1 expressed in mammalian cells, thus confirming the presence of post-translational modifications on Pdx1 when expressed in mammalian cells.

### Endogenous Pdx1 also Exist in Multiple Isoforms

**Figure 3:**
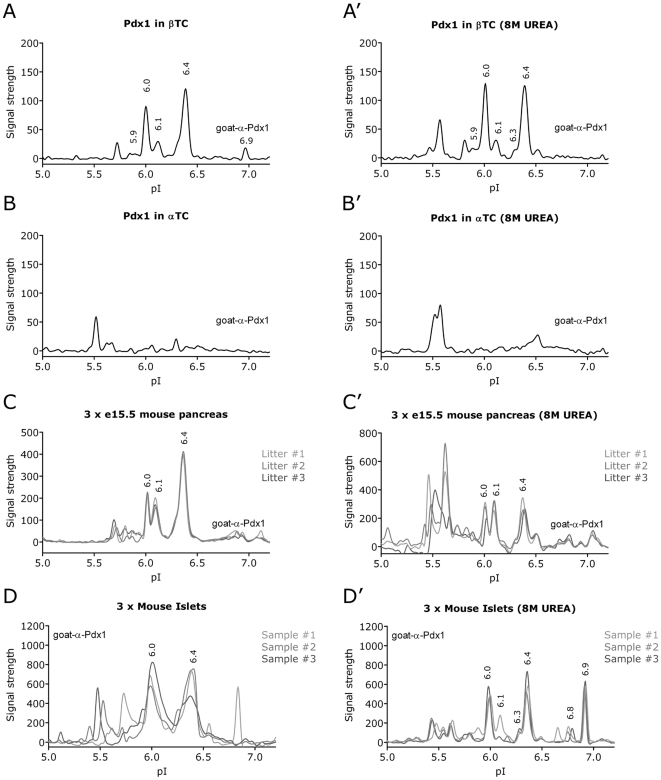
The NIA profile of endogenous Pdx1. Analysis of cells with know endogenous expression of Pdx1. As negative control we included αTC cells which express little or no Pdx1. To rule out the possibility of protein-protein complexes affecting the NIA profile, the lysates were diluted 20 fold in a mild Hepes buffer (HNG) (A-D) or in 8 M urea (A’-D’) prior to NIA analysis. Using the goat-α-Pdx1 antibody, the characteristic 6.0, 6.1 and 6.4 peaks and to a lesser extend also the 6.3 peak is observed in βTC (A, A’) but not in αTC (B, B’). To show the reproducibility of the NIA assay between the HNG and 8 M urea we compared the Pdx1 profile from e15.5 mouse pancreas obtained from three different litters (C, C’) and from 3 different preparations of purified mouse islets (D, D’). We also performed these analyses with the mouse-α-Pdx1 antibody (Fig. S2), confirming the dominant peaks at 6.0, 6.1 and 6.4 as well as the islet enriched 6.9 peak.

Having confirmed that we are able to detect multiple isoforms of Pdx1 following over-expression in mammalian cell lines we went on to test if the NIA analysis could be used to detect endogenous Pdx1 protein. We therefore analyzed the Pdx1 profile from various cell types and tissues known to be positive or negative for Pdx1 expression. Another question we wanted to address was to what extent the Pdx1 profile is influenced by protein-protein interactions. Normally, the NIA analysis is conducted in a mild Hepes buffer (HNG) and it is therefore possible that some of the Pdx1 peaks we observe represent a protein complex. We therefore compared the profiles of endogenous Pdx1 in the HNG buffer (Fig. 3A-D) with that obtained in 8 M urea which will denature most interactions (Fig. 3A’-D’). According to the manufactures instruction urea treatment gives better resolution but reduces signal intensity. Using the goat-α-Pdx1 antibody on βTC cells the characteristic 6.0, 6.1 and 6.4 peaks were clearly seen in both buffers (Fig. 3A, 3A’). To a lesser extend also the 5.9 peak and perhaps the 6.3 as a faint shoulder on the left side foot of the 6.4 peak. The 6.9 peak was small but significantly present in HNG buffer only, whereas the 6.5 peak was only observed in the urea treated samples. The peak at 5.8 which was only detected with the goat-α-Pdx1 antibody was present in both buffers while the non-pdx1 peak at 5.6 was only observed with urea. In αTC cells which express little or no Pdx1, the strongest peaks were at pI 5.5 and 5.6 and perhaps also at 6.5 in urea treated lysate (Fig. 3B, 3B’). In embryonic tissue Pdx1 is expressed in the pancreatic endoderm and in three independent lysates from isolated embryonic pancreata we observed a profile very similar to βTC with the strongest peaks at pI 6.0, 6.1 and 6.4 (Fig. 3C, 3C’). This observation was very reproducible in both buffers although again the peaks around 5.6 became more prominent in 8M urea. Lastly, we compared the profile obtained from three independent samples of isolated mouse islets (Fig. 3D, 3D’). Here we observed increased variation between the different samples, but interestingly in the urea treatment revealed a strong peak at pI 6.9 in addition to the characteristic 6.0 and 6.4 peaks. We also detected some smaller peaks around 5.9, 6.1 and 6.8. Lastly, we analyzed the different samples with the mouse-α-Pdx1 antibody (Fig. S2), confirming the peaks at 6.0, 6.1 and 6.4 as well as the islet enriched 6.9 peak.

In summary the NIA analysis of three cell types known to express endogenous Pdx1 produced several peaks which varied slightly between the different cell types. However, the overall profile is very similar to that obtained using over-expression.

### Both Endogenous and Exogenous Pdx1 Carry One Principal Phosphorylation

**Figure 4:**
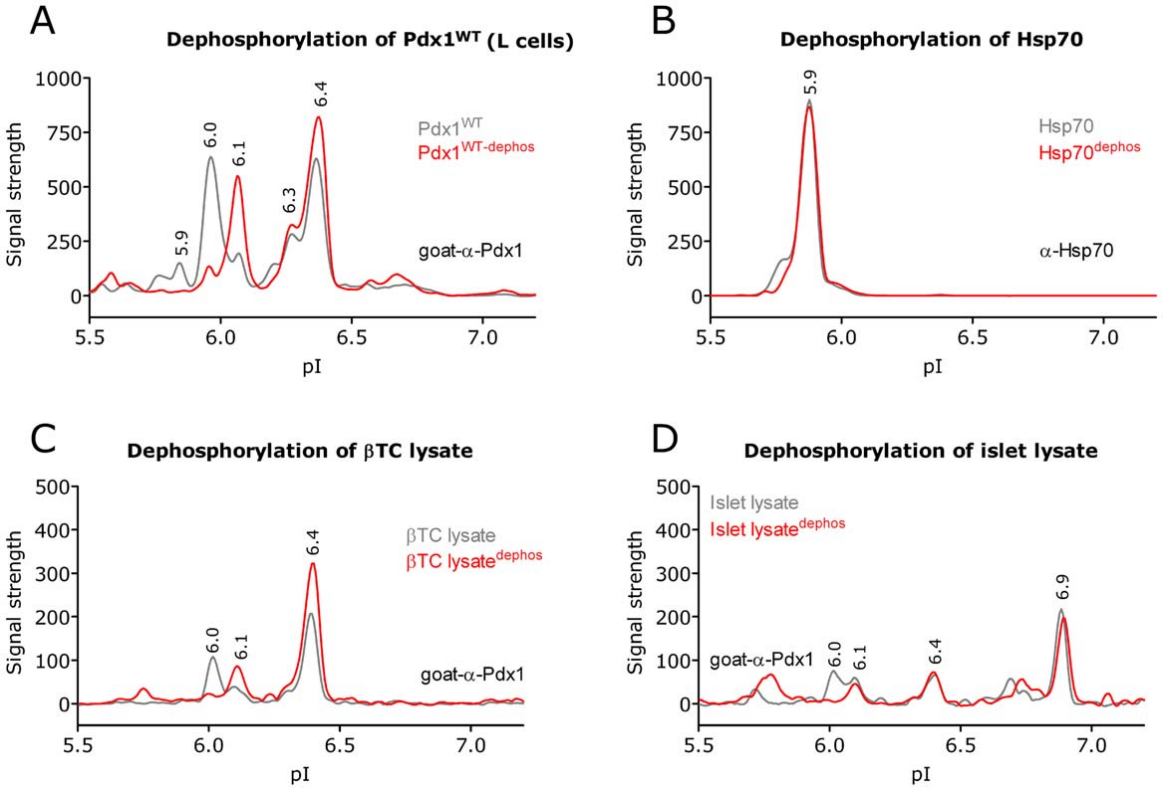
Pdx1 is phosphorylated. In order to determine the identity of the peaks found in the Pdx1 profile we treated the lysate with lambda phosphatase to see if the removal of phosphorylations would shift the peaks. A-D) NIA profile (in 8 M urea) of the dephosphorylated lysate (red) is show superimposed on the control treated lysate (grey). A) Over expression of pdx1^WT^ in L results in a shift of the 6.0 peak to 6.1, which fits the expected change in pI caused by a phosphorylation. The 6.40 peak is unaffected by the dephosphorylation. B) The NIA profile of Hsp70 from the same lysates serves as a control to show that the dephosphorylation assay does not impact the profile of a non phosphorylated protein. Control treatment or dephosphorylation of βTC cells (C) and mouse islets (D), show similar results. Results are representative of at least three independent experiments.

Depending on expression levels and cell types we detect at least four or five differentially charged isoforms of Pdx1 in various cell types and tissues while bacterially generated Pdx1 only produces a single peak. This strongly suggests that Pdx1 is subject to post translational modifications in mammalian cells. Numerous studies have shown that Pdx1 is phosphorylated [25], [26], [27], [28], [29], [30], [31], [32], [33], [34] and to test this, L cells were transfected with Pdx1 and the lysates were subsequently incubated with or without Lambda phosphatase and subjected to NIA analysis. The NIA profile from non-treated and phosphatase treated lysates were superimposed and revealed that the phosphatase treatment shifted the 6.0 peak to 6.1 while the 6.3 and 6.4 peaks remained unaffected (Fig. 4A). This suggests that Pdx1 harbors one primary phosphorylation represented by the 6.0 peak. The smaller peaks at 5.9 are also affected by the phosphatase treatment and it is likely that they represent additional but less abundant phosphorylations. To validate that the phosphatase treatment *per se* did not affect the NIA profile we analyzed the same lysates for the endogenous protein Hsp70 (Fig. 4B) and found the Hsp70 profiles for treated verses non-treated to be identical. Similar results were observed in βTC (Fig. 4C) and mouse islets (Fig. 4D) which express endogenous Pdx1.

### Serine 61 is the Primary Site of Phosphorylation in Pdx1

**Figure 5:**
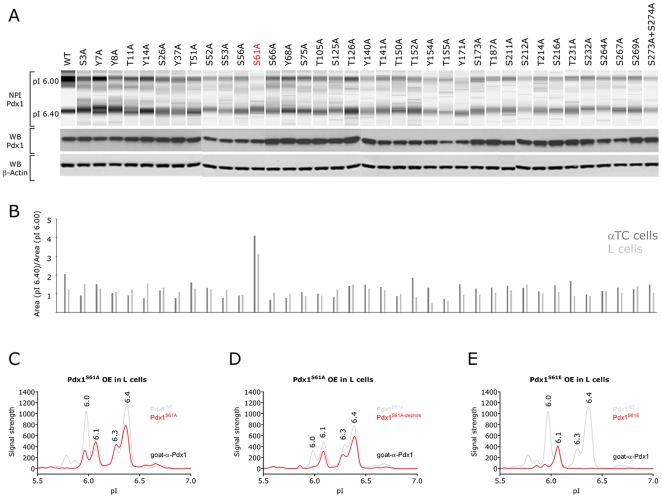
NIA profiles of Pdx1 alanine scanning shows Pdx1 to primarily be phosphorylated on serine 61. To identify putative phosphorylation sites in Pdx1, which may not have been picked up by the MS, we carried out an alanine scan. A) All serines, tyrosines and threonines in Pdx1 were mutated into alanines and transfected into L cells and αTC cells which are negative for endogenous Pdx1 expression and subjected to NIA analysis. B) Western blots against Pdx1 and β-Actin were used to confirm the expression of Pdx1 from the plasmids. Since the 6.4 peak is unaffected by dephosphorylation we used the area under curve (pI 6.4)/area under curve (pI 6.0) ratio to identify mutants where the phosphorylation was reduced. In most cases the intensity of the two bands are similar resulting in a relative ratio around 1 in both L cells (light grey bars) and αTC cells (dark grey bars). However, in Pdx1^S61A^ the phosphorylated band (pI 6.0) was reduced and as a result the relative ratio increased to around 4. The screen was performed twice in each cell line and a representative result is shown. C-D) NIA profiles of S61 mutants (red) superimposed on the wild type Pdx1 profile (grey) (C). Also, dephosphorylation of the S61A mutant (red) superimposed on the control-treated lysate (grey), shows a residual peak at 6.0 which is removed by phosphatase (D).Mutating S61 to the phospho-mimic glutamic acid (E) clearly reduces the 6.0 peak. Note, that the entire Pdx1 profile of this mutant including the 6.3 and 6.4 peaks are moved to the left, as would be expected from the calculated pI change of an alanine to glutamic acid substitution. The NIA analysis in C-E were done in both L and αTC (data not shown) cells in least three independently transfected cell lysates, yielding similar results.

To test if the NIA assay could be used to map the phosphorylated residue in Pdx1 we carried out an alanine scan where all serines, tyrosines and threonines which are putative phosphorylation sites were replaced by alanine. Plasmids encoding the mutated Pdx1 proteins were transfected into L cells and αTC cells. The lysates were analyzed using NIA or western blots to confirm the expression and SDS-PAGE mobility of Pdx1 (Fig. 5A). To estimate the amount of phosphorylated Pdx1 we took advantage of the observation that the signal intensity of the 6.4 peak is unaffected by phosphorylation while the intensity of the 6.0 peak is related to the amount of phosphorylated protein. The ratio between the 6.4 and the 6.0 peak should therefore reflect the relative proportion of phosphorylated Pdx1. We find that only Pdx1^S61A^ differs markedly from wild type Pdx1 (Fig. 5B) and superimposing the profile of Pdx1^S61A^ onto the wild type profile revealed a marked reduction of the 6.0 peak (Fig. 5C). However, a residual 6.0 peak which could be removed by phosphatase treatment could still be detected, indicating the presence of at least one additional phosphorylation (Fig. 5D). To examine how constitutive phosphorylation affects the NIA profile of Pdx1 we replaced serine 61 with the negatively charged residue glutamic acid (Pdx1^S61E^), hereby mimicking a phosphorylation. When Pdx1^S61E^ was analyzed using the NIA assay we only observed a single peak at pI 6.1(Fig. 5E) suggesting that the modifications of Pdx1 are absent if the protein is phosphorylated. This might also explain why the 6.3 and 6.4 peaks are unaffected following de-phosphorylation.

### Mass Spectrometry Analysis of Pdx1

**Figure 6:**
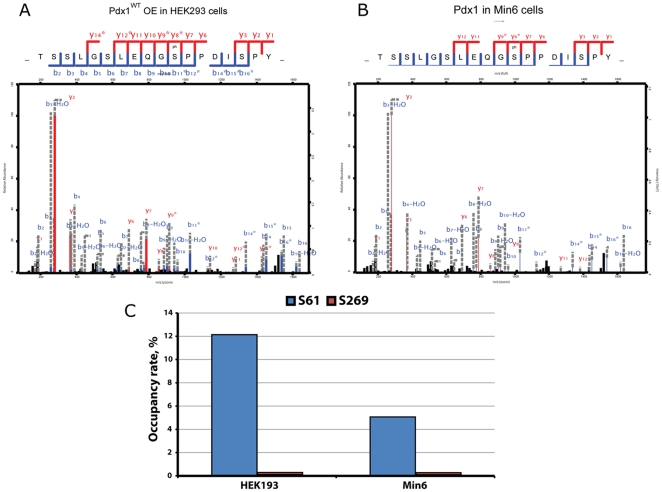
MS analysis identifies serine 61 as the principal phosphorylation site in Pdx1. To substantiate the NIA data we did mass spectometry to identify modified residues especially looking for phosphorylations. We analyzed endogenous Pdx1 protein from Min6 cells and over expressed Pdx1 from HEK293 cells. The mouse wildtype Pdx1 protein was, purified by immunoprecipitation using the mouse-α-Pdx1 antibody, then separated by SDS-PAGE, digested with chymotrypsin and subjected to MS analysis. A-B) MS/MS data showing phosphorylation on wildtype Pdx1 at serine 61 when over experessed and purified from HEK293 cells (A) or from Min6 cells (B). We then did a quantification of the amount of phosphorylated versus non-phosphorylated Pdx1 peptides counted by the MS instrument. C) Relative amount of phosphorylated serine 61 and serine 269 in HEK293 and Min6 cells. The MS analysis was done once.

The NIA analysis of Pdx1 identified S61 as the primary phosphorylation of Pdx1 in L cells, αTC cells and HEK293 cells (data not shown). To confirm this finding we purified Pdx1 protein from over-expressing HEK293 cells or from Min6 cells which express endogenous Pdx1. The purified protein was initially digested with trypsin. However, despite a 98% coverage of the Pdx1 protein, we were not able to conclusively detect the S61 phosphorylation. In order to investigate this discrepancy in more detail we analyzed Pdx1 under chymotryptic treatment prior to mass spectrometric analysis. Using chymotrypsin ensures that Pdx1 is proteolytically cleaved at different amino acids (Phe, Ile, Tyr and Trp) as compared to when using trypsin (Lys and Arg). This allows for increased sequence coverage of the analyzed protein and hence increased analytical coverage of PTMs. Using this strategy we were able to significantly confirm the presence of S61 phosphorylation in both HEK293 cells (Fig. 6A) and in Min6 cells (Fig. 6B). To evaluate the occupancy rate for the identified phosphorylation sites we compared the peptide abundance of unmodified peptide to its phosphorylated counterpart (Fig 6C). Although this is not the most accurate measure it still provides information as to how extensive a given amino acid is phosphorylated. The comparison reveals that S61 indeed is the most highly abundant phosphorylation form in comparison to S269, with a ∼12% S61 occupancy rate in HEK293 and a corresponding S61 occupancy rate of ∼5% in Min6 cells. In comparison the occupancy rates for S269 phosphorylation in both HEK293 and Min6 were found to be less than 1%.

### The Pdx1 Profile is Remarkably Stable

**Figure 7:**
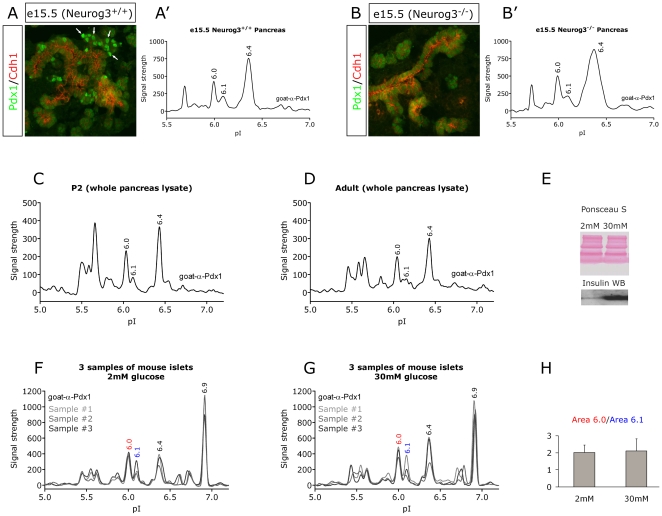
The Pdx1 NIA profile from *in vivo* β-cells is not unique and not responsive to glucose. To determine if the β-cells contain a unique Pdx1 protein species we compared the NIA profiles of pancreata obtained from wild type mice and mice lacking the pro-endocrine gene *Neurog3*. In the *Neurog3^−/−^* mice all β-cells are lost and the Pdx1 high expressing β-cells are no longer present. Thus, any modifications on Pdx1 that are uniquely present in the β-cell should therefore only appear in the wild type profile. A and B) Immunohistochemical stainings of e15.5 *Neurog3^+/+^* or *Neurog3^−/−^* mouse pancreata, showing the distribution of Pdx1 (green) in the Chd1 positive endoderm (red). A’ and B’) the Pdx1 NIA analysis of equivalent micro dissected tissue. Pdx1 NIA profile of pancreas tissue lysate from newborn (P2) (C) and adult NMRI mice (D). Results are representative of two (Neurog3) or three independent (P2 and adult) experiments. E, F and G) Three independent purifications of islets from NMRI mice were cultured for 2–3 days, washed with media lacking glucose, and then cultured with 2 mM glucose for two hours followed by incubation with 2 mM or 30 mM glucose for one hour E) Western blot on media from the islets, showing glucose responsiveness. F) Three preparations of mouse islets in 2 mM glucose. G) Three preparations of mouse islets in 30 mM glucose. H) Quantification of the three experiments showing the ratio of the pI 6.0 peak area under curve divided by the pI 6.1 peak area under curve. The error bars show standard deviation.

Several studies have shown that Pdx1 is modified in response to glucose stimulation and oxidative stress [26], [27], [28], [29]. To test if the endogenous Pdx1 profile is dependent on cellular context or exogenous stimulation we proceeded to investigate the NIA profile of several sources of β-cells. In the embryonic pancreas the developing β-cells can be distinguished from other Pdx1 expressing cells because they express higher levels of Pdx1 (Fig. 7A; white arrows). In mice lacking the pro-endocrine gene Neurog3 the endocrine lineage is absent and as a result the high expressing Pdx1 cells are no longer be detected (Fig. 7B). To test if the Pdx1 profile from the high expressing β-ell population has any unique features we dissected and homogenized whole pancreata from wild type and Neurog3 deficient e15.5 embryos and subjected the lysates to NIA analysis. As for all other cell types analyzed we detected the 6.0, 6.1 and 6.4 peaks and we could not detect any obvious differences between the wild type and the pancreata lacking β-cells (Fig. 7A’, 7B’). Next we examined the NIA profile from pancreas lysates of newborn (Fig. 7C) and adult (Fig. 7D) mice pancreata to see if any changes could be associated with the β-cells becoming functional and being exposed to fluctuating blood glucose levels. At both P2 and in adult mice the 6.0, 6.1 and 6.4 peaks were indistinguishable from the previously observed Pdx1 profiles including embryonic tissue suggesting that the Pdx1 profile is relatively stable after birth. To test if changes in glucose levels had any effect on the NIA profile we isolated mouse islets and incubated them at low or high glucose (2 mM and 30 mM) for 1 hour, before harvest. We confirmed that the islets were glucose responsive by measuring the amount of insulin released to the media using western blotting (Fig. 7E). However, the NIA analysis shows the profiles from three independent experiments in 2 mM or 30 mM glucose, respectively (Fig. 7F, 7G). It is evident that the profiles obtained from high and low glucose is remarkably similar and when the ratio between the 6.0 peak and the 6.1 peak is calculated there is no difference between the two conditions (Fig. 7H).

### Mouse Pdx1 is Modified in the Developing Endoderm of Mouse and Chicken Embryos

**Figure 8:**
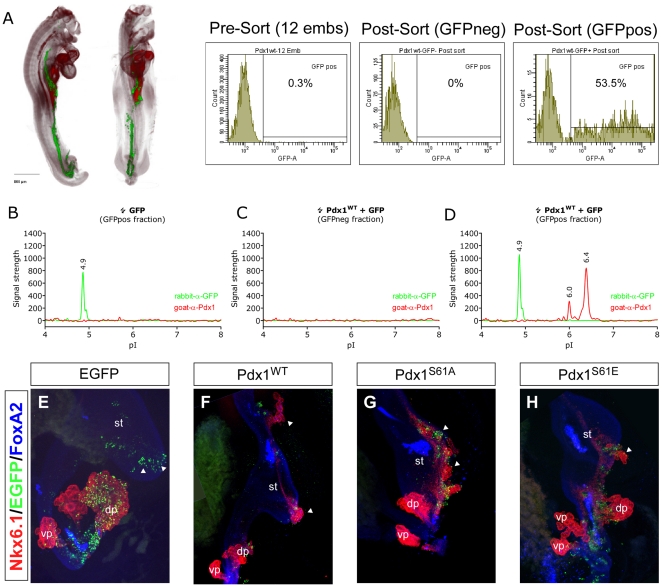
Pdx1 is phosphorylated in the developing chicken endoderm but S61 phosphorylation is not required for ectopic pancreas formation. Since the characteristic Pdx1 NIA profile was found early in mouse endoderm development (Fig. S3), we investigated the function of Pdx1 by *in ovo* electroporation of plasmids encoding Pdx1 and GFP into the endoderm of developing chicken embryos. One day following electroporation GFP expressing cells could be detected throughout the endoderm. A) OPT images showing the distribution of GFP expressing cells (green). Following dissociation, the GFP expressing cells were purified using FACS. Before sorting, the GFP expressing cells constituted 0.3% of the total number while the sorted fraction contained 53.5% GFP cells. B-D) The NIA profiles of the GFP positive fraction of embryos only electroporated with GFP (B), GFP and Pdx1 in the GFP negative fraction from embryos electroporated with GFP and Pdx1 (C) and the GFP positive fraction from embryos electroporated with both GFP and Pdx1 (D). In embryos electroporated with wild type Pdx1 the profile resembles the canonical Pdx1 profile. E-H) 3D image projections of confocal sections obtained from chicken embryos stained for Nkx6.1 (red), GFP (green) and Foxa2 (blue) by whole mount immunohistochemistry 72 hours after electroporation. In embryos electroporated with plasmids encoding GFP no ectopic Nkx6.1 expression could be detected (E). In embryos electroporated with wild type Pdx1 we observed the induction of ectopic pancreata and associated Nkx6.1 expression (F; arrowheads). Plasmids encoding Pdx1^S61A^ (G) and Pdx1^S61E^ (H) and also induced ectopic pancreata when electroporated into the endoderm. We also observed that the ectopic pancreata induced by wild type Pdx1 and S61A was associated with ectopic insulin expression (Fig. S4). dp (dorsal pancreas), st (stomach). Results are representative for two independent FACS analyses and at least three wholemount stainings for each Pdx1 construct.

In addition to its role in the β-cell Pdx1 is also required for pancreas development during embryogenesis. At e10.5 Pdx1 is expressed in both the dorsal and ventral pancreatic buds, in the duodenum and in the posterior stomach (Fig. S3A). The stomach and pancreas region from wild type embryos was micro dissected and the pooled pancreata from two litters were analyzed by NIA (Fig. S3A’). The signal obtained is weak but we could robustly detect the 6.0, 6.1 and 6.4 peaks indicating that Pdx1 is modified during the earliest stages of pancreas development. Slightly later in development at e12.5 the pancreas express high levels of Pdx1 while low Pdx1 expression can be detected in the stomach and duodenum (Fig. S3B). Again we found the distinctive Pdx1 profile albeit at higher levels (Fig. S3B’). To test if the phosphorylated form of Pdx1 is important for pancreas development we electroporated mouse Pdx1 into the endoderm of living chicken embryos using *in ovo* electroporation [44]. Using this system Pdx1 would be expressed in an environment closely resembling the developing pancreas and presumably be exposed to the similar signaling cues. One day after electroporation GFP expression can be detected throughout the endoderm (Fig. 8A). The embryos were then dissociated into single cells and sorted into GFP negative and GFP positive fractions using FACS. On average GFP cells constituted 0.3% of the total number of cells (pre-sort) and 53% after sorting. Each embryo contained 5000–10000 GFP expressing cells. Running NIA analysis on cells from embryos electroporated only with GFP, the GFP sorted fraction was positive for GFP but not Pdx1 (Fig. 8B), showing that we do not detect endogenous chicken Pdx1. In the NIA analysis of embryos co-electroporated with GFP and Pdx1 neither of the two proteins were detected in the GFP negative fraction (Fig. 8C), but only in the GFP positive fraction (Fig. 8D). As expected, the Pdx1 profile produced peaks at pI 6.0 and pI 6.4 confirming that Pdx1 becomes phosphorylated in the developing endoderm. Finally, we electroporated chicken embryos with plasmids encoding GFP, Pdx1^WT^, Pdx1^S61A^ or Pdx1^S61E^. The embryos were allowed to develop for 3 days, before being dissected and subjected to immunohistochemistry. The embryos were analyzed for the expression of GFP, Foxa2 (expressed in all endodermal cells) and Nkx6.1. Nkx6.1 is a marker of pancreas development and has previously been shown to be induced by Pdx1 [7], [8]. In embryos electroporated with GFP alone the pancreas and anterior endoderm developed normally (Fig. 8E). Importantly, no expression of Nkx6.1 was detected in the endoderm outside the pancreatic buds. When Pdx1^WT^ was electroporated we observed a strong induction of ectopic Nkx6.1 expression in the endoderm anterior to the pancreas (Fig. 8F). To investigate if the S61 phosphorylation has a role in pancreas development we looked for ectopic activation of Nkx6.1 expression in chicken embryos electroporated with Pdx1^S61A^ and Pdx1^S61E^ (Fig. 8G, 8H). We found that both mutants induces pancreas budding and Nkx6.1 expression to exactly the same extent as the wildtype and it is therefore unlikely that phosphorylation of S61 has a role in pancreas development. In embryos electroporated with Pdx1^WT^ the ectopic Nkx6.1 was found scattered within the Foxa2 positive endoderm. However, in several cases we observed that the Nkx6.1 cells became organized into a branched epithelium very similar to the endogenous pancreas (arrows in Fig. 8F-8H). We therefore speculated that the ectopic Nkx6.1 expression might actually reflect the formation of ectopic pancreata. To test this we stained embryos electroporated with Pdx1^WT^ or Pdx^1S61A^ for GFP, Nkx6.1 and insulin. In all embryos where we could detect ectopic Nkx6.1 expression anterior to the pancreas we also observed insulin expression (Fig. S4A and S4B).

## Discussion

One advantage of the NIA technology is that one antibody is used for the detection of all protein species which can be separated by electrical charge. In recent years NIA has been used successfully to study signal transduction [41], [42] but, to the best of our knowledge this work represents the first study where the technology has been applied to a transcription factor. When analyzing the profile of the homeobox containing transcription factor Pdx1 we find at least four differently charged protein species Pdx1^6^.^0^, Pdx1^6^.^1^, Pdx1^6^.^3^ and Pdx1^6^.^4^. These data were obtained using two different antibodies against Pdx1 and we were able to shift the peaks by adding an epitope tag. Furthermore, the four peaks are always present when Pdx1 is expressed in eukaryotic cells (βTC, mouse islets, mouse embryonic pancreas, L cells, HEK293, αTC and chicken endoderm cells) while bacterially derived Pdx1 only produces a single peak. Given the similar SDS-PAGE mobility of eukaryotic and bacterial Pdx1 we conclude that the NIA peaks we observe are due to post-translational modifications. Also, as both eukaryotic and bacterially expressed Pdx1 gives a single band using western blots we conclude that the post-translational modifications must be relatively small, i.e. under 1000 Da.

One very important aim of this study was to determine the pI of bacterially expressed and hereby minimally modified Pdx1 protein and to this end confirm that the Pdx1 protein is in fact subjected to post-translational modification in eukaryotic cells. We did this by comparing the NIA profiles of Pdx1 purified from bacteria and mammalian cells. As expected the bacterial protein produced a single peak. However, the bacterial peak has a pI of 7.2 while the predicted pI for unmodified mouse Pdx1 is 6.4. In lysates from eukaryotic cells the most prominent peak actually runs at 6.4, importantly when comparing bacterial and mammalian Pdx1 we used exactly the same open reading frame for expression and we sequence verified the different expression vectors and can conclusively rule out any possibility of errors in the constructs or the presence of alternative start sites. We therefore conclude that the pI for unmodified Pdx1 is pI 7.2 and that it is a coincidence that the 6.4 peak observed in eukaryotic cells has the same pI as predicted for unmodified Pdx1. This is supported by NIA analysis of human pdx1, which has a predicted pI of 7.1, but when over expressed in HEK293 cells gives the dominant peak at pI 6.6 (data not shown). Interestingly, on western blots Pdx1 also behaves differently from what would be expected. The predicted molecular weight of Pdx1 is 31 kDa but on gels the protein migrates at 40–46 kDa. This difference has now been proven by mass spectrometry to be due to anomalous SDS-PAGE mobility of the pdx1 protein [45] and we speculate that similar anomalous properties of Pdx1 in isoelectric focusing is the reason for the difference between observed and predicted pI of bacterially expressed Pdx1 protein.

Using the NIA assay and mass spectrometry we identified S61 as the primary phosphorylation and that the modification is responsible for the 6.0 peak. However, the mass spectrometry also identified phosphorylations on Y140/T141, S269 and in the NIA profile we could still detect a smaller peak at 6.0 in the S61A mutant that could be shifted following de-phosphorylation. To test if Y140/T141 orS269 could account for the residual phosphorylation in Pdx1^S61A^ we generated the following double mutants Pdx1^S61A/Y140A^, Pdx1^S61A/T141A^ Pdx1^S61A/S269A^. However, all the double mutants showed low levels of phosphorylation (data not shown). Given the numerous publications describing other phosphorylation sites on Pdx1 it is obvious that any of those could be present together with the S61 phosphorylation.

Given the dynamic and often fast exchange of phosphorylation and post-translational modifications in general it is remarkable that there is so little variation between Pdx1 obtained from the very different cell types. Both endogenous and ectopically derived Pdx1 produces profiles where the 6.0 and 6.4 peaks represent the predominant protein species. Subtle variations might be found, for example the 6.1 peak appears to be more abundant in the E15.5 embryonic tissue. However, only in isolated islets do we observe a significantly different peak at pI 6.9. This peak is interesting for several reasons. First, it is only observed following treatment with 8 M urea indicating that in HNG buffer this Pdx1 species is masked from antibody detection. A likely explanation is that that the 6.9 species only exists in a tight protein-protein complex. Second, the 6.9 peak is only found in isolated islets strongly suggesting that it is unique to mature beta-cells. It should be noted that that the 6.9 peak, was absent in post-natal and adult pancreas, but this is probably due to the expression of Pdx1 in acinar cells, masking the Pdx1 signal from the relatively few β-cells present in the adult mouse pancreas [46].

What our data clearly shows is that the level of glucose has no impact the NIA profile of Pdx1 from purified mouse islets. This is in agreement with other studies also using non-invasive detection techniques to study Pdx1 protein from both mouse, human and rat islets [29], [45]. One explanation for this could be that the factors modifying Pdx1 are ubiquitous. For example if the posttranslational modifications of Pdx1 were coupled to common events such as the cell cycle [47]. Alternatively, ubiquitous factors known to interact with Pdx1 such as p300 [48] might be present in all cell types and therefore influence the profile in a similar manner.

One important feature of the NIA assay is that can be used to study how the different modifications influence each other. Although we fail to identify the modification responsible for the 6.3 and 6.4 peaks it is very obvious that these peaks are uncoupled from the phosphorylation of S61. Thus the de-phosphorylation only affects the 6.0 and 6.1 peaks. Furthermore, S61E which mimics a constitutive phosphorylation abolished other protein isoforms. One explanation for this could be that the modification responsible for the 6.3/6.4 peaks only can occur when S61 is not phosphorylated.

Likewise the residual phosphorylation peak (6.0) in Pdx1^S61A^ is remarkably larger than what would be expected if the secondary phosphorylation peak (5.9) of the wild type Pdx1 protein was shifted to (6.0). Thus interactions between different modifications or redundant phosphorylation sites are likely to influence the dynamics of Pdx1 modification.

In conclusion, we show that Pdx1 harbors several post-translational modifications and that phosphorylation of serine 61 is the most abundant phosphorylation on Pdx1, and that it is not regulated by glucose in mouse islets.

## Materials and Methods

### Nanofluidic Proteomic Immunoassay (NIA)

This assay, here designated NIA, was developed by Cell Bioscience [42]. All runs were done on the Nanopro1000 instrument and performed essentially as described by the manufacturer (http://www.cellbiosciences.com/nanopro.html). The NIA assay is a fully automated, nanocapillary-based immunoassay based on isoelectric focusing (IEF) to separate the proteins in a regular cell or tissue lysate. Following separation the proteins are linked to the capillary wall through UV irradiation and then probed with primary and secondary antibodies. The secondary antibody is HRP-labeled, which enables sensitive chemiluminescence detection. The signal, which is black dots equivalent to that obtained by western blotting are then analyzed by special software and can be viewed as either a graph or a virtual gel. All NIA data presented in this study were obtained by duplicate analysis of the described lysates in either HNG buffer (20 mM Hepes, pH7.5, 25 mM NaCl, 10% glycerol) or 8 M urea, both supplemented with 20 mM DTT. This was done to compare the NIA profile under conditions of semi- and full-denaturation. Samples were then mixed 1∶1 with a sorbitol based premix, containing five peptide standards with known pI (pI ladder 1 cat.no. 040–644). The same premix was used for all runs and it enables the generation of a pH gradient ranging from 4 to 9 (cat.no. 040–319). To establish the pH gradient, the anolyte were 10 mM phosphoric acid and the catholyte were 100 mM sodium hydroxide. The proteins were focused for 40 min. at 15 mW, and then incubated for 2 hours with primary antibody; 4 times wash with TBST and 1 hour with the secondary HRP conjugated antibody, washed again and lastly, chemiluminescence detection with SuperSignal West Dura from Pirece. Concerning peak identification we observe small variations from run to run. The peak at pI 6.40 could therefore vary from 6.36 to 6.44. However, the relative distance between peaks in the same run was always the same.

Dephosphorylation was done *in vitro* using Lambda phosphatase (NEB, Cat.no. P0753). Cell lysate were diluted 10 times in reaction buffer (50 mM HEPES, 100 mM NaCl, 2 mM DTT, 4 mM MnCl_2_), split in two and treated with 1 µl (400 units) lambda phosphatase or 1 µl water for 30 min. at 37°C. Afterwards the reactions were diluted once in HNG or 8 M urea, both with 20 mM DTT and analyzed by NIA as described above.

### MS Analysis

All MS experiments were performed on a nanoscale HPLC system (EASY-nLC from Proxeon Biosystems) connected to a hybrid LTQ–Orbitrap Velos (Thermo Fisher Scientific) equipped with a nanoelectrospray source (Proxeon Biosystems). Each peptide sample was auto-sampled and separated in a 15 cm analytical column (75 µm inner diameter) in-house packed with 3-µm C18 beads (Reprosil Pur-AQ, Dr. Maisch) with a 2 h gradient from 5% to 40% acetonitrile in 0.5% acetic acid. The MS instrument was operated in data-dependent mode to automatically switch between full-scan MS and MS/MS acquisition. Survey full-scan MS spectra (from m/z 300–1,700) were acquired in the orbitrap with resolution R  =  30,000 at m/z 400 (after accumulation to a ‘target value’ of 1,000,000 in the linear ion trap) using a HCD top10 method. The ten most intense peptide ions with charge states ≥2 were sequentially isolated to a target value of 50,000 using predictive automatic gain control (pAGC) and fragmented by higher-energy collisional dissociation (HCD) in the octopole collision cell using normalized collision energy of 40%. The ion selection threshold was 5,000 counts for HCD and the maximum allowed ion accumulation times were 500 ms for full scans and 250 ms for HCD. All HCD fragment ion spectra were recorded in the orbitrap with a resolution of 7,500 at m/z 400. For all full scan measurements a lock-mass ion from ambient air (m/z 445.120025) was used for internal calibration when present, as described [49]. Standard mass spectrometric conditions for all experiments were: spray voltage, 2.2 kV; no sheath and auxiliary gas flow; heated capillary temperature, 200°C; predictive AGC with calibrated scaling factor of about 100, and an S-lens RF level of 60%.

### Identification of Peptides and Proteins by MaxQuant

The data analysis was performed with the MaxQuant software (version 1.0.14.7, www.maxquant.org) as described [50] supported by Mascot (www.matrixscience.com) as the database search engine for peptide identifications. We followed the step-by-step protocol of the MaxQuant software suite [51] to generate MS/MS peak lists that were filtered to contain at most six peaks per 100 Da interval and searched by Mascot (Version 2.2.04) against a concatenated target/decoy (forward and reversed) version of the IPI human database version 3.37 (69.316 forward protein entries). Protein sequences of common contaminants such as human keratins and proteases used were added to the database. The initial mass tolerance in MS mode was set to 7 p.p.m. and MS/MS mass tolerance was 0.02 Da. Cysteine carbamidomethylation was searched as a fixed modification, whereas lysine acetylation, serine/threonine/tyrosine phosphorylation, protein N-acetylation, and oxidized methionine were searched as variable modifications. A maximum of two mis-cleavages was allowed while we required strict tryptic specificity. The resulting Mascot.dat files were loaded into the MaxQuant software together with the raw data for further analysis. To minimize false identifications, all top-scoring peptide assignments made by Mascot were filtered based on previous knowledge of individual peptide mass error. Peptide assignments were statistically evaluated in a Bayesian model on the basis of sequence length and Mascot score. We accepted peptides and proteins with a false discovery rate of less than 1%, estimated on the basis of the number of accepted reverse hits.

### Chicken Electroporations and FACS Analysis

Chicken electroporations was performed as previously described [44]. Wholemount immunefluorescence staining on mouse and chicken embryos were performed as previously described [52]. For OPT analysis embryos were stained and scanned on an OPT scanner 3001 M (Bioptonics, Edinburgh UK) according to manufacturers instructions. For FACS all embryos were electroporated with 10 µg of pCGIG5 vector containing the gene for cytoplasmic EGFP as well as 10 µg of either empty vector or mouse Pdx1^WT^. The embryos were harvested 24 hours after electroporation by digestion with 300 µl 1% trypsin per 2 embryos for 10 min. at 37°C, with 3 rounds of gentle pipeting. The trypsin were neutralised with DMEM media containing 10% FBS. After centrifugation at 1200×g for 5 min. the cells from 12 embryos were resuspended in app. 7 ml FACSflow buffer and sorted by EGFP fluorescence on a FACSaria instrument (BD Bioscience). The resulting fractions were either EGFP negative, app. 1.5 million cells or EGFP positive, app. 50.000 cells. These fractions were centrifuged and the EGFP negative fractions lysed in 100 µl lysis buffer and the EGFP positive fractions in 4 µl. For details on the lysis buffer please refer to the supplementary information.

### Cell lines, Islets and Embryonic Tissue

βTC3 (βTC), αTC1.3 (αTC), L and HEK293F (HEK293) cells were cultured in DMEM with 10% FBS and 1% pen/strep. βTC, L and HEK293 cells were cultured with 5 g/L glucose, and αTC with 1g/L glucose. Islets from wild type NMRI mice were isolated using collagenase digestion and were grown for 5 days in RPMI 1640 with 10%FBS and 1% pen/strep. All embryonic mouse tissue were from wild type NMRI mice, expect for the Neurog3-knockout [53]. For analysis of embryonic pancreas tissue time-mated mice were sacrificed at the indicated gestational stage, the embryos were removed and the embryonic pancreas dissected under microscope using a small knife and forceps.

### Cloning and Plasmids

Wildtype mouse Pdx1 (Pdx1^WT^) cDNA were obtained as an imageclone from Open Biosystems (GenBank: BC103581.1). This cDNA was amplified by PCR and inserted in the pENTR/D vector (Invitrogen) and then transferred to our previously published expression vector pCGIG5 [44], for expression in eukaryotes and to the pEXP2 plasmid (Invitrogen) for expression in bacteria. To generate, the 3xFlag version we used a 3’ primer comprising the tag. To introduce point mutations we performed chimeric PCR using primers covering 12 nucleotides up and down stream of the codon being modified. Correct clones were identified by diagnostic RE digests and sequence verified (MWG, Ebersberg, Germany). As control we used the pCGIG5 vector either without insert (designated empty vector) or with EGFP. Wildtype GSK3β were cloned from Xenopus and a kind gift from Chris Wright.

### Cell/tissue Harvest

For direct NIA and western analyzes all cell lines, tissue and islets were lysed in Tissue Extraction Reagent I (50 mM Tris, pH 7.4, 250 mM NaCl, 5 mM EDTA, 2 mM, Na_3_VO_4_, 1 mM NaF, 20 mM Na_4_P_2_O_7_, 0.02%NaN_3_, and proprietary detergent), from Invitrogen. Prior to use this buffer was supplemented with protease inhibitors (Complete protease inhibitor cocktail tablets, Roche). Before harvest, the cells were washed once in PBS and thoroughly aspirated. One 6-well of cells was lysed in app. 100 µl, one litter of E15.5 pancreata was lysed in 20 µl and 200 islets in 8 µl. For the Neurog3-KO, individual pancreata were isolated and snap-frozen in dry ice and kept at −80°C, while genotyping was performed. Pancreata with the three different genotypes was pooled and lysed in 5 µl buffer per four pancreata. HEK293 lysates for immunoprecipitation followed by NIA, western or mass spectrometry analysis were harvested as nuclear extracts; One 15 cm dish were washed once in PBS followed by cytolysis for 10 min. in 1.5 ml buffer A (10 mM HEPES pH 7.9, 10 mM KCl, 0.1 mM EDTA and 0.4% Triton X-100, Complete protease inhibitor cocktail tablets). Then, scraped and dissociated by pippeting and centrifuged at 10.000xg for 5 min. The pellet was resuspended in 300 µl buffer B (20 mM HEPES pH 7.9, 400 mM NaCl, 2 mM EDTA and 20% glycerol, Complete protease inhibitor cocktail tablets), sheared using a 21 G needle and shaken for 1 hour at 4°C. To generate the bacterially expressed protein, 30 ml of BL21 overnight culture was added to 250 ml fresh media and grown for 3 hours before induction with 1 mM IPTG for additionally 6 hours. The resulting bacteria pellet was washed and resuspended in 12 ml TBST supplemented with protease inhibitors, followed by sonication on ice.

Additionally, all lysates were kept on ice for at least 10 min, with 2 rounds of vortexing, followed by centrifugation at 16.000×g for 20 min. and then transferred to clean tubes and kept at −80°C.

### Immunoprecipitation (IP) and Western Blotting (WB)

For IP of wildtype Pdx1, BL21 lysates and nuclear extracts from HEK293 cells transfected with empty vector or Pdx1^WT^ were incubated with 5 µg mouse-α-Pdx1 antibody and 50 µl magnetic protein G dynabeads (Invitrogen) per ml of lysate, for 3 hours at 4°C with end-over turning. Following four washes in TBST the beads were eluted in 8 M urea supplemented with 20 mM DTT at 30°C for 60 min with shaking. For IP of 3xFlag tagged Pdx1, BL21 lysates and nuclear extracts from HEK293 cells transfected with empty vector or Pdx1^3xFLAG^ were incubated with 25 µl magnetic α-Flag beads (Sigma) per ml of lysate for 3 hours at 4°C with end-over turning. Following four washes in TBST the 25 µl beads were eluted with 125 µl of 100 µg/µl 3xFlag peptide in TBS at 37°C for 60 min with shaking. Lastly, all eluates were concentrated app. 20 fold using spin columns with a 10 K cut-off (Amicon).

Pdx1 protein for mass spectrometry was prepared from nuclear extracts of HEK293 cells transfected with Pdx1^WT^ and immunoprecipitated as described above, however elution from the beads were done by heating at 80°C with LDS sample buffer (Invitrogen) containing 20 mM DTT. Following SDS-PAGE, the gel was stained with Coomassie (Imperial stain, Pierce); the band of interest cut out, destained with ammonium sulphate and sliced into small pieces; then reduced with DTT and alkylated using chloroacetamide. Finally, the proteins were digested overnight with trypsin, loaded onto StageTips and subjected to mass spectrometric analysis.

Western blots were done using precast NuPAGE Novex Bis-Tris gels and blotted onto PVDF membranes (Invitrogen). All membranes were stained with Ponceau S to verify uniform blotting. Destaining was done with 5% acetic acid. Blocking and antibody diluent were 5% skim milk with 0.1% tween-20. The antibodies were applied for 1 hour at room temp or overnight at 4°C. Detection was done with Supersignal west pico from Pierce. Afterwards, when appropriate the membranes were incubated overnight in skim milk with 0.02% sodium azide and then reproped with another primary antibody of a different species.

### Cell line Transfections

For transfection of αTC and L cells, plasmid DNA was purified from 40 ml overnight TOP10 bacteria culture using the S.N.A.P. kit from invitrogen. All pointmutants were transfected into each cell line in at least two different cell passages. This was done in 6- or 12-well plates using Lipofectamine2000 according to manufactures instructions. For transfection of HEK293 cells, plasmid DNA was isolated from 600 ml overnight culture using the Megaprep kit, from Invitrogen. HEK293 cells were transfected with empty vector or Pdx1^WT^ in 15 cm dishes using Lipofectamine2000 according to manufactures instructions.

### Antibodies

Primary goat-α-Pdx1 was a kind gift from Chris Wright and used at 1:50.000 for western, 1∶10.000 for immunofluorescence and between 1∶50 and 1∶500 for NIA. Primary mouse- α-Pdx1 from the Hybridoma bank (1 µg/µl) was used at 1∶10.000 for western, 1∶5–1∶50 for NIA and for IP at 5 µg/ml lysate. Rabbit-α-GFP from Invitrogen were used for NIA at 1∶50 and mouse-α-Hsp70 from Abcam was used for NIA at 1∶1.000. For western, mouse-α-β-Actin from Sigma was used at 1∶25.000, guine a pig-α-insulin from Abcam at 1∶100 and mouse-α-GSK3α/β from R&D at 1∶500. For immunofluorescence; rabbit-α-GFP from Clonetech was used at 1∶500, mouse-α-Nkx6.1 from the Hybridoma bank at 1∶1000 and goat-α-FoxA2 from Santa Cruz at 1∶250. All secondary antibodies were from Jackson Immunoresearch, the cross absorbed versions. For western and NIA they were HRP conjugated and used at 1∶10.000 (western) or 1∶500 (NIA). For immunofluorescence they were Cy2, Cy3 or Cy5 conjugated and used at 1∶300.

## Supporting Information

Figure S1 **Spiking of Pdx1^3xFLAG^ purified from bacteria into HEK293 cells expressing either EGFP^Flag^ or Pdx1^3xFlag^.** To further validate that the pI of bacterially expressed Pdx1 does differ from that of Pdx1 expressed in the human HEK293 cell line, we mixed the purified Pdx1^3xFLAG^ protein sample from BL21 bacteria into HEK293 lysate expressing either EGFP^FLAG^ or Pdx1^3xFLAG^ and then ran the NIA assay. As control we spiked HEK293 lysates expressing (A) EGFP^FLAG^ or (B) HEK293 lysates expressing Pdx1^3xFLAG^ with a negative control sample consisting of bacterial proteins purified from untransformed bacteria. As expected from Fig.3 we only observed the Pdx1^3xFLAG^ profile. (C and D) Then we spiked the same two HEK293 lysates with purified bacterially expressed Pdx1^3xFLAG^ and it is clear that the profile was not altered by mixing the samples before doing the NIA analysis. Two analyses were performed giving similar results.
(TIF)

Figure S2 **NIA profile of endogenous Pdx1 using the mouse-α-antibody.** Samples were diluted in 8 M urea and analyzed by NIA with the mouse-α-Pdx1 antibody. This was done in parallel to the profiles obtained with the goat-α-Pdx1 antibody, presented in figure 3. A-D) NIA analysis showing the profile of βTC (A), αTC (B), E15.5 pancreas (C) and islets (D) obtained with the mouse-α-Pdx1 antibody (red) superimposed on the profile obtained from the same samples using the goat-α-Pdx1 antibody (grey). Two analyses were performed giving similar results.
(TIF)

Figure S3 **The Pdx1 protein is detected in developing mouse endoderm.** Immunohistochemical stainings showing the Pdx1 expression (green) in the Chd1 positive endoderm (red). At e10.5 (A) and e12.5 (B) Pdx1 is expressed uniformly in the pancreas, posterior stomach and in the duodenum. A’ and B’) Pdx1 NIA analysis of equivalent micro dissected tissue, showing that during very early development at e10.5 Pdx1 also appears to show the characteristic NIA profile and two days later at e12.5 the profile is easily recognizable. Results are representative of three independent experiments.
(TIF)

Figure S4 **Both Pdx1^WT^ and Pdx1^S61A^ induces ectopic insulin expression** ***in vivo*** **.** 3D image projections of confocal sections obtained from chicken embryos stained for Insulin (red), Nkx6.1 (blue) and GFP (green) by whole mount immunohistochemistry 72 hours after electroporation. In embryos electroporated with plasmids encoding (A) Pdx1^WT^ or (B) Pdx1^S61A^ we observe an equal amount of ectopic insulin production. Two analyses were performed giving similar results.
(TIF)
