# Supplementary figures and images for: Pdx1 Is Post-Translationally Modified In vivo and Serine 61 Is the Principal Site of Phosphorylation
Source: PLoS One. 2012 Apr 11;7(4):e35233. doi: 10.1371/journal.pone.0035233 (PMC3324462; doi:10.1371/journal.pone.0035233)

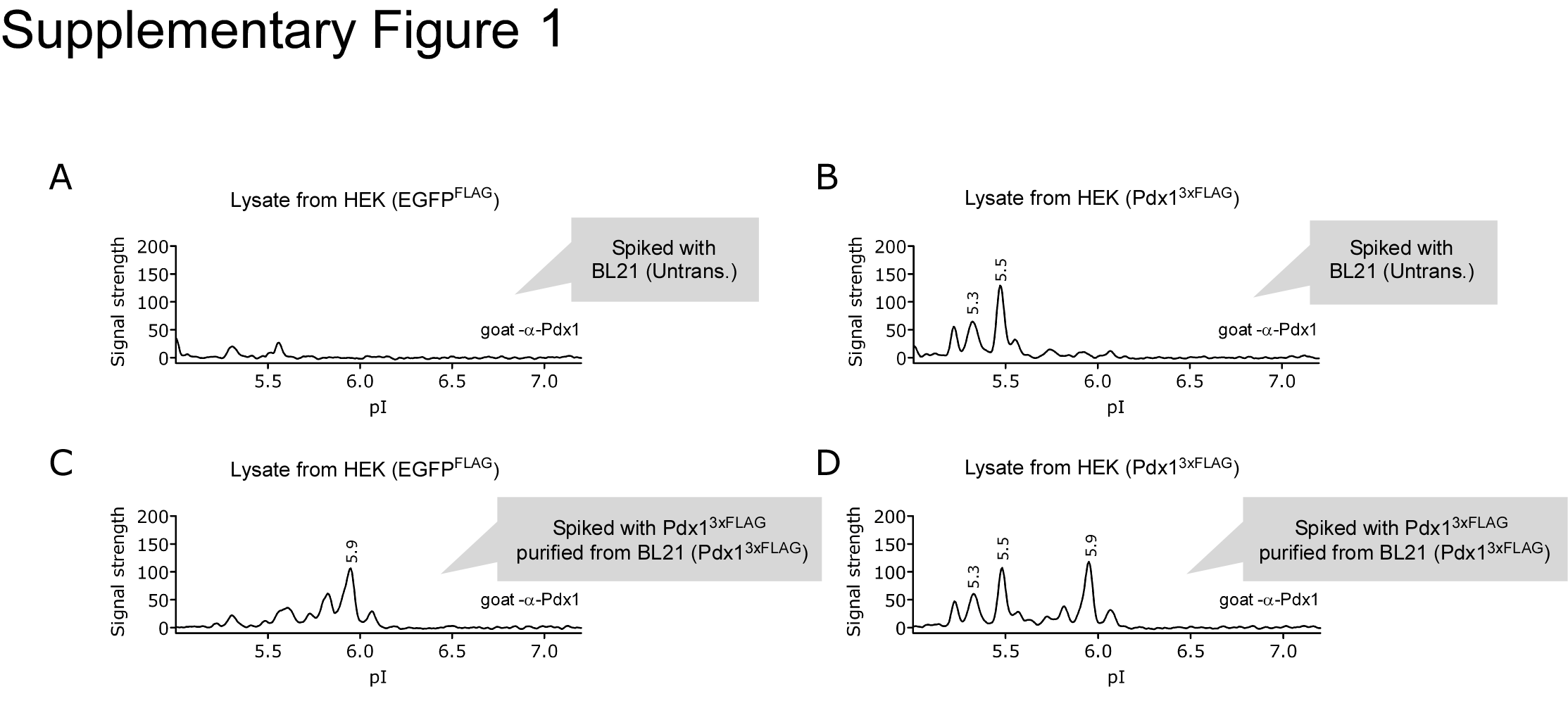

Supplement: Figure S1 — Spiking of Pdx13xFLAG purified from bacteria into HEK293 cells expressing either EGFPFlag or Pdx13xFlag. To further validate that the pI of bacterially expressed Pdx1 does differ from that of Pdx1 expressed in the human HEK293 cell line, we mixed the purified Pdx13xFLAG protein sample from BL21 bacteria into HEK293 lysate expressing either EGFPFLAG or Pdx13xFLAG and then ran the NIA assay. As control we spiked HEK293 lysates expressing (A) EGFPFLAG or (B) HEK293 lysates expressing Pdx13xFLAG with a negative control sample consisting of bacterial proteins purified from untransformed bacteria. As expected from Fig.3 we only observed the Pdx13xFLAG profile. (C and D) Then we spiked the same two HEK293 lysates with purified bacterially expressed Pdx13xFLAG and it is clear that the profile was not altered by mixing the samples before doing the NIA analysis. Two analyses were performed giving similar results. (TIF) [file pone.0035233.s001.tif]

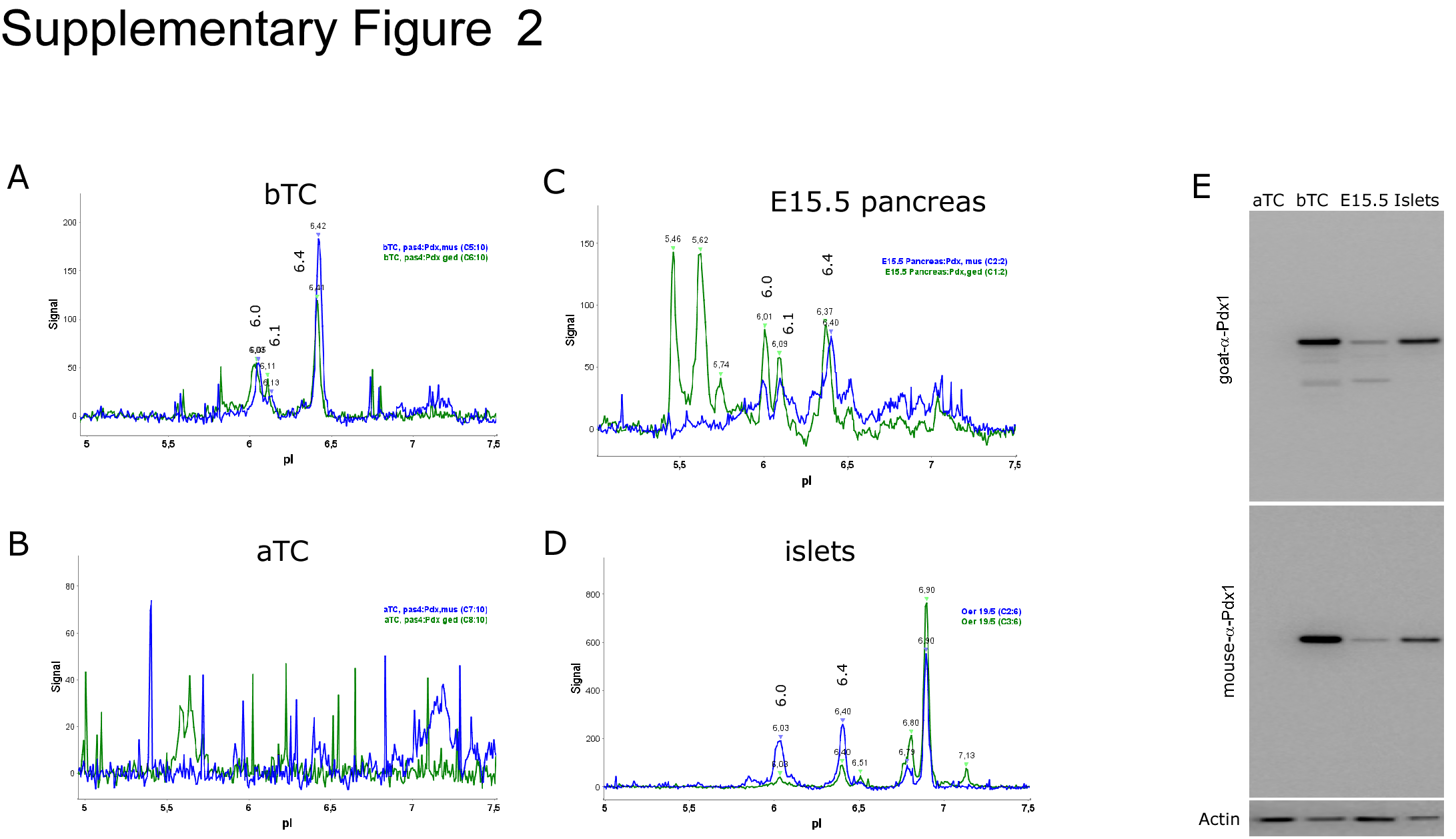

Supplement: Figure S2 — NIA profile of endogenous Pdx1 using the mouse-α-antibody. Samples were diluted in 8 M urea and analyzed by NIA with the mouse-α-Pdx1 antibody. This was done in parallel to the profiles obtained with the goat-α-Pdx1 antibody, presented in figure 3. A-D) NIA analysis showing the profile of βTC (A), αTC (B), E15.5 pancreas (C) and islets (D) obtained with the mouse-α-Pdx1 antibody (red) superimposed on the profile obtained from the same samples using the goat-α-Pdx1 antibody (grey). Two analyses were performed giving similar results. (TIF) [file pone.0035233.s002.tif]

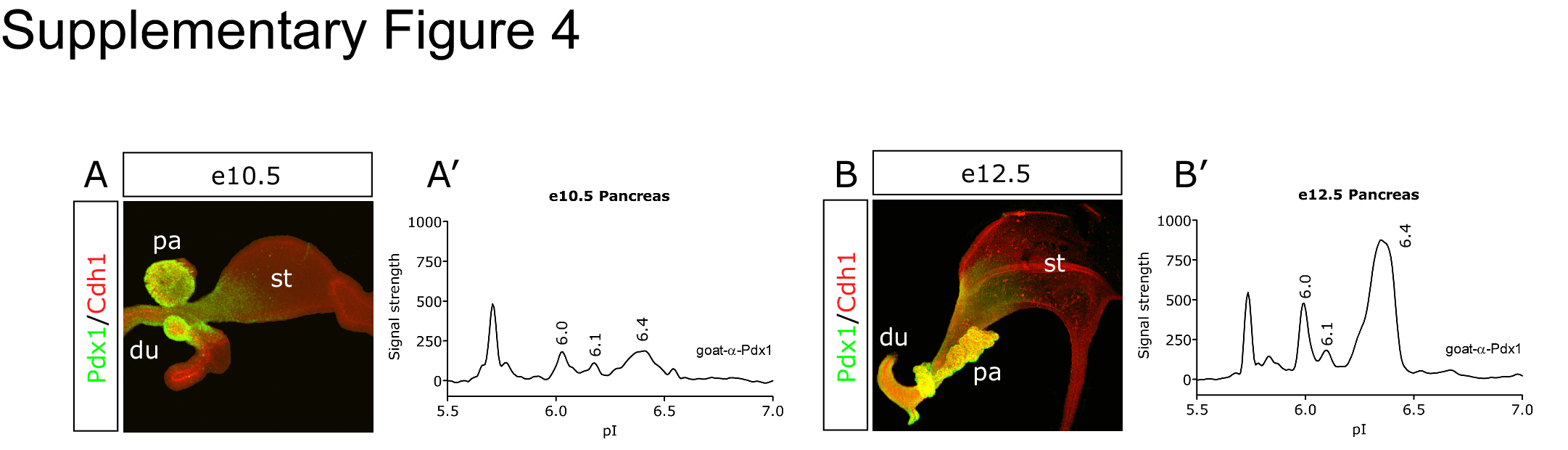

Supplement: Figure S3 — The Pdx1 protein is detected in developing mouse endoderm. Immunohistochemical stainings showing the Pdx1 expression (green) in the Chd1 positive endoderm (red). At e10.5 (A) and e12.5 (B) Pdx1 is expressed uniformly in the pancreas, posterior stomach and in the duodenum. A’ and B’) Pdx1 NIA analysis of equivalent micro dissected tissue, showing that during very early development at e10.5 Pdx1 also appears to show the characteristic NIA profile and two days later at e12.5 the profile is easily recognizable. Results are representative of three independent experiments. (TIF) [file pone.0035233.s003.tif]

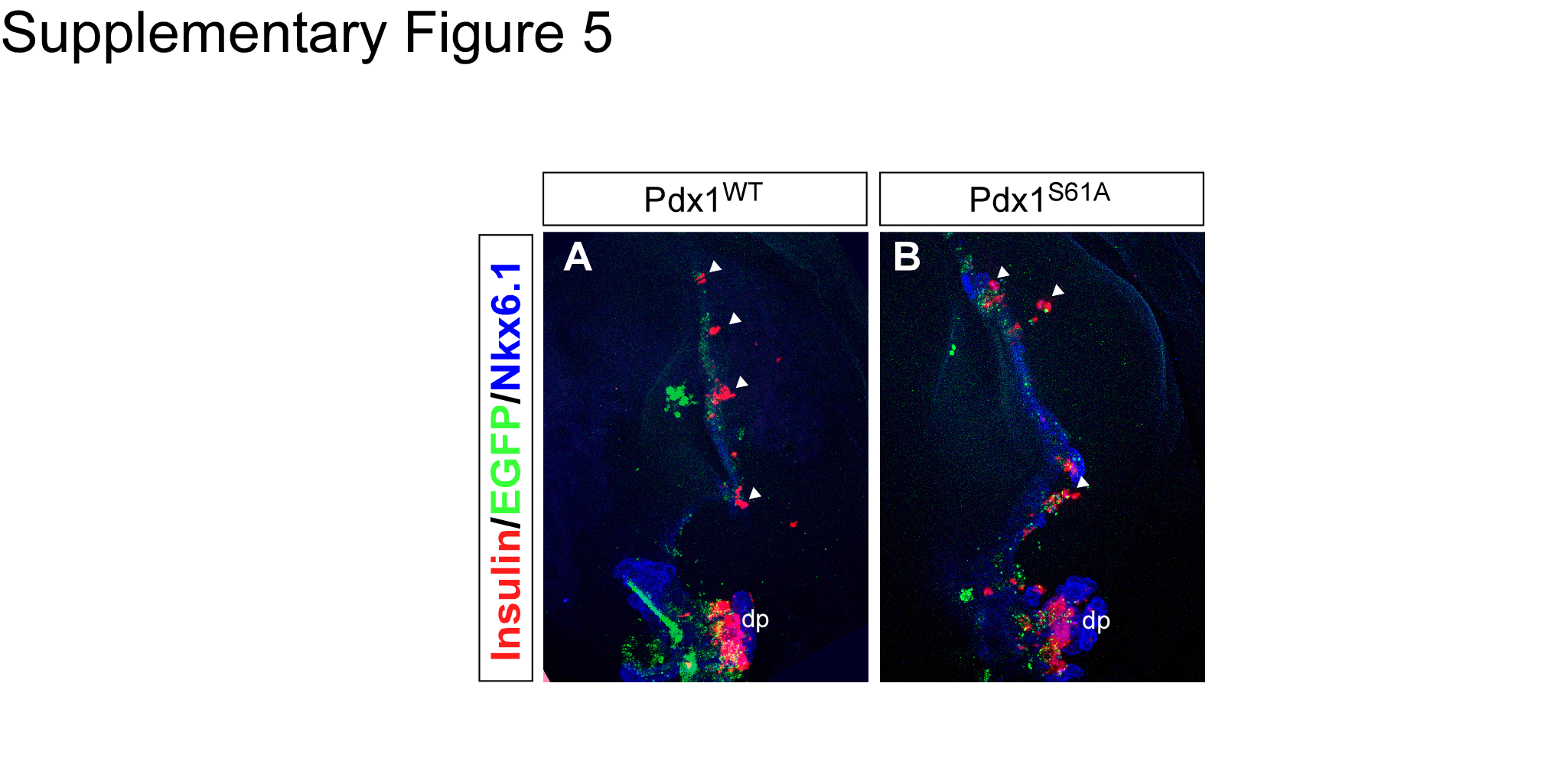

Supplement: Figure S4 — Both Pdx1WT and Pdx1S61A induces ectopic insulin expression in vivo . 3D image projections of confocal sections obtained from chicken embryos stained for Insulin (red), Nkx6.1 (blue) and GFP (green) by whole mount immunohistochemistry 72 hours after electroporation. In embryos electroporated with plasmids encoding (A) Pdx1WT or (B) Pdx1S61A we observe an equal amount of ectopic insulin production. Two analyses were performed giving similar results. (TIF) [file pone.0035233.s004.tif]
